# Editorial: Comparative animal consciousness

**DOI:** 10.3389/fnsys.2022.998421

**Published:** 2022-10-19

**Authors:** Louis N. Irwin, Lars Chittka, Eva Jablonka, Jon Mallatt

**Affiliations:** ^1^Department of Biological Sciences, The University of Texas at El Paso, El Paso, TX, United States; ^2^Research Centre for Psychology, Queen Mary University of London, London, United Kingdom; ^3^Cohn Institute for the History of Philosophy of Science and Ideas, Tel Aviv University, Tel Aviv-Yafo, Israel; ^4^School of Biological Sciences, Washington State University, Pullman, WA, United States

**Keywords:** animal cognition, animal awareness, vertebrates, arthropods, cephalopods, definition of consciousness

The scientific study of consciousness has seen a resurgence in the 21st century. This collection of reviews, essays, and theories on various aspects of comparative animal consciousness takes a biological and evolutionary approach. As defined here, consciousness refers to the process by which an animal has *perceptual and affective experience or feelings, arising from the material substrate of a nervous system*. It draws upon a long tradition of neuroscientific materialism (Jackson, 1887; Churchland, 1986, 2013; Dennett, 1991; Feinberg, 2012) and a recent emphasis on neurophenomenology (Varela, 1996; Gallagher and Zahavi, 2008; Tononi and Koch, 2015; Irwin and Irwin, 2020; Seth, 2021).

The implications of evolutionary theory for the continuity of life inevitably extended investigations of consciousness to species other than humans. Darwin (1871) believed that consciousness is an evolved capacity, shaped by natural selection and graded in complexity. Arguments for its widespread distribution and ancient origins come from various lines of evidence, including documentation of a variety of different but sufficiently complex, hierarchical neural architectures (Tononi and Edelman, 1998; Dehaene and Naccache, 2001; Dehaene and Changeux, 2011; Barron and Klein, 2016; Feinberg and Mallatt, 2016; Ginsburg and Jablonka, 2019; Carvalho and Damasio, 2021; Chittka, 2022), discovery of sensitivity to stimuli undetectable by humans (Chittka, 2017), behavioral indicators of emotion and self-awareness (Mather, 2008; Baars and Edelman, 2012; Paul et al., 2020; Mallatt et al., 2021; Chittka, 2022), evidence for the adaptive role of associative learning and declarative memory (Bronfman et al., 2016; Ginsburg and Jablonka, 2019), and the cognitive capability for place perception and control of movement (Merker, 2005; Engel, 2010; Chittka and Wilson, 2019; Irwin and Irwin, 2020). Taken together, these studies have led to a growing but not unanimous view that all vertebrates, many arthropods, and cephalopods meet these criteria for sensory and affective consciousness, indicating that consciousness evolved independently in arthropods and vertebrates over half a billion years ago, followed by the cephalopods later in the Paleozoic (Barron and Klein, 2016; Feinberg and Mallatt, 2016; Ginsburg and Jablonka, 2019; Godfrey-Smith, 2020). Alternative theories have been advanced focusing on mechanisms that likely restrict consciousness to birds, mammals, and some reptiles (Humphrey, 1992; Butler and Cotterill, 2006; Edelman et al., 2011; Pennartz et al., 2019; Nieder et al., 2020) or even to humans alone (Chaisson, 1987; LeDoux, 2019). This collection seeks to shed light on this range of views.

If consciousness arose independently in at least three different clades with very different neural architectures, how many different evolutionary trajectories to consciousness are theoretically possible? The long-standing “multiple realizability thesis” maintains that since so many neural architectures exist across the animal kingdom, the same mental states can arise from an almost unlimited number of different architectures (Putnam, 1967). Mallatt and Feinberg argue that, while different architectures can give rise to different forms of consciousness, the forms that mental states can take are not unconstrained. Since consciousness emerged under the influence of the same vital stimuli (temperature, odors, sounds, electromagnetic waves, etc.) some similarity in neuroanatomy and perceptual content is required in order for different taxa to survive in competition in the same physical world. At the same time, others emphasize that as evolutionary pressures differ profoundly between species, so do their sense organs, perceptual systems, and mental operations (Bräuer et al., 2020; Montemayor, 2021; Chittka, 2022).

Thurston Lacalli reasons that consciousness evolved like all biological attributes, from simple antecedents that were progressively elaborated and refined over an extended period of evolutionary time as stepwise adaptations in different cognitive niches. In his first contribution to this volume, Lacalli focuses on “selector circuits” of neurons that encode irreducible elements of experience (qualia) as subunits of the neural correlates of consciousness that evolve through progressive refinement. In his second contribution, Lacalli envisions how distinctive qualia evolved from more diffuse and less differentiated “original (ur-) qualia.”

Two articles on how natural selection can channel consciousness toward greater complexity are included here. Tjøstheim et al. note that navigation, including taking detours, appears to be an essential element of consciousness, because it requires map-like cognitive structures for spatial representation beyond the animal's immediate location. By using simulations in a forced detour paradigm, they show how different strategies can yield behaviors that approximate those of different species. They propose that both neuronal population size and inhibitive efficacy may be important for allowing organisms to negotiate predation risks and natural geometries that obstruct foraging.

In the second example,Van De Pol and van Swinderen build on the paradoxical view of brain function as an ongoing balance between prediction and surprise as a factor in understanding the evolution of consciousness. In particular, this view may provide insight into the function and evolution of active sleep, which is widespread in animals, not just in mammals and birds. They suggest that such sleep evolved as a mechanism for refining and generalizing internal models of the world during sleep, to minimize prediction errors in the waking state.

The earliest vertebrates to evolve were jawless fishes. Suzuki reviews the evidence that the surviving members of that clade — lampreys and hagfish — display the markers of primary, minimal consciousness. He concludes that the adult lamprey appears to meet the neuroanatomical criteria for mediating consciousness. While less is known about hagfish, their sensory behaviors and learning abilities are more amenable to lab testing, and may soon provide the basis for conclusions about their capacity for consciousness as well.

Molluscs, with mostly small brains or merely dispersed ganglia, separated from the lineage to vertebrates over 550 million years ago. But cephalopods soon diverged as a molluscan subgroup, evolving large nervous systems, complex behavior, and significant cognitive abilities (Young, 1964; Grasso and Basil, 2009; Schnell and Clayton, 2021). In a wide-ranging review of historical and current research on the neuroanatomy, behavior, and cognitive abilities of cephalopods, Ponte et al. conclude from five different criteria that these animals have the capacity for at least a basal faculty of consciousness. They further advocate for asking, not “Is this species more conscious than that one?” but rather, “How is the individual experience of this species different from that one?” Kaufmann endorses that formulation, pointing to the growing realization that placing an organism on a single sliding-scale model for consciousness is a methodological mistake. Rather, the behavioral, cognitive and neurological criteria for conscious experience should be sensitive to experience-specific differences conceived within a multidimensional framework that provides a distinct consciousness profile for each species. An example of a non-linear multidimensional model gaining traction is one proposed by Birch et al. (2020).

The paper by Carls-Diamante questions the common notion that consciousness must have a unified structure by noting that a majority of the neurons in an octopus are found, not in its brain, but in its arms. She raises the intriguing idea that each octopus arm may be capable of supporting its own idiosyncratic field of consciousness, limited in content to the sensory and motor processes relevant to that arm. She then points out that if we are to have a more comprehensive understanding of different types of creature consciousness, particularly among invertebrates, we need to go beyond vertebrate-based assumptions about phenomenal experience, such as the notions that there is only one conscious field per organism and that only the CNS can generate conscious fields.

Numerous authors have viewed motility as a primary driver for the evolution of consciousness (Sheets-Johnstone, 1999; Merker, 2005; Engel, 2010; Chittka and Wilson, 2019). Vallortigara likewise makes the core assumption that animals have evolved phenomenal experience in strict association with active movement. Here and in previous writing (Vallortigara, 2020), he invokes the concept of the internally-generated efference copy to distinguish between sensations (what is happening to me, internally) from perceptions (what is happening out there, externally), as originally proposed by Humphrey (1992). Vallortigara argues that consciousness arises from the interplay of this internally-generated efference copy and sensory input from the outside world.

Problem solving through insight may be another window into animal consciousness. Though difficult to investigate in non-verbal animals, Osuna-Mascaro and Auersperg suggest that it may be widespread and amenable to study through proxy indicators, such as eye-tracking, pupil dilation, and emotionality.

Michael Levin provides an overarching perspective that places animal consciousness as a process within a broader population of “cognitive systems,” and invites a reconsideration of the traditional limited conceptions of cognition, the self, memory, regeneration, developmental programs, and evolution. His article provides many novel insights, including questions of agency, the nature of the Self, an expansive view of intelligence, the operation and architecture of distributed memory, and various aspects of consciousness.

The net effect of the contributions to this volume is to support the growing acceptance of the idea that consciousness is ancient in origin and widespread across the phylogenetic spectrum, arising in a diversity of nervous systems, and manifested in a variety of ways (Darwin, 1871; Koch, 2012; Feinberg and Mallatt, 2016, 2018; Chittka and Wilson, 2019; Ginsburg and Jablonka, 2019; Irwin, 2020). They also point to the need for a definition of consciousness, like the one proposed in the first paragraph of this editorial, that is generic enough to encompass a broad range of animal phenomenologies.

## Author contributions

LI wrote the draft version of the paper and oversaw revisions. LC, EJ, and JM made extensive suggestions of both an editorial and substantive nature. All authors have read and approved the final version.

## Conflict of interest

The authors declare that the research was conducted in the absence of any commercial or financial relationships that could be construed as a potential conflict of interest.

## Publisher's note

All claims expressed in this article are solely those of the authors and do not necessarily represent those of their affiliated organizations, or those of the publisher, the editors and the reviewers. Any product that may be evaluated in this article, or claim that may be made by its manufacturer, is not guaranteed or endorsed by the publisher.

## References

[B1] Baars B. J. Edelman D. B. (2012). Consciousness, biology and quantum hypotheses. Phys. Life Rev. 9, 285–294. 10.1016/j.plrev.2012.07.001 22925839

[B2] Barron A. B. Klein C. (2016). What insects can tell us about the origins of consciousness. Proc. Natl. Acad. Sci. U S A. 113, 4900–4908. 10.1073/pnas.1520084113 27091981 PMC4983823

[B3] Birch J. Schnell A. K. Clayton N. S. (2020). Dimensions of animal consciousness. Trends Cogn Sci. 24, 789–801. 10.1016/j.tics.2020.07.007 32830051 PMC7116194

[B4] Bräuer J. Hanus D. Pika S. Gray R. Uomini N. (2020). Old and new approaches to animal cognition: There is not “one cognition”. J. Intell. 8, 28. 10.3390/jintelligence8030028 32630788 PMC7555673

[B5] Bronfman Z. Z. Ginsburg S. Jablonka E. (2016). The transition to minimal consciousness through the evolution of associative learning. Front. Psychol. 7, 1954. 10.3389/fpsyg.2016.01954 28066282 PMC5177968

[B6] Butler A. B. Cotterill R. M. (2006). Mammalian and avian neuroanatomy and the question of consciousness in birds. Biol. Bull. 211, 106–127. 10.2307/4134586 17062871

[B7] Carvalho G. B. Damasio A. (2021). Interoception and the origin of feelings: A new synthesis. Bioessays. 43, e2000261. 10.1002/bies.202000261 33763881

[B8] Chaisson E. (1987). The Life Era: Cosmic Selection and Conscious Evolution. Boston, MA: Atlantic Monthly Press.

[B9] Chittka L. (2017). Bee cognition. Curr. Biol. 27, R1049–R1053. 10.1016/j.cub.2017.08.008 29017035

[B10] Chittka L. (2022). The Mind of a Bee. Princeton, NJ: Princeton University Press.

[B11] Chittka L. Wilson C. (2019). Expanding consciousness. *Amer*. Scientist 107, 364–369. 10.1511/2019.107.6.364

[B12] Churchland P. M. (2013). Matter and Consciousness (3rd ed.). Cambridge, MA: MIT Press.

[B13] Churchland P. S. (1986). Neurophilosophy. Cambridge: MIT Press.

[B14] Darwin C. (1871). The Descent of Man (2nd ed.). New York: A. L. Burt.

[B15] Dehaene S. Changeux J. P. (2011). Experimental and theoretical approaches to conscious processing. Neuron 70, 200–227. 10.1016/j.neuron.2011.03.018 21521609

[B16] Dehaene S. Naccache L. (2001). Towards a cognitive neuroscience of consciousness: basic evidence and a workspace framework. Cognition 79, 1–37. 10.1016/S0010-0277(00)00123-2 11164022

[B17] Dennett D. C. (1991). Consciousness Explained. Boston: Little, Brown and Co.

[B18] Edelman G. M. Gally J. A. Baars B. J. (2011). Biology of consciousness. Front. Psychol. 2, 4. 10.3389/fpsyg.2011.00004 21713129 PMC3111444

[B19] Engel A. K. (2010). “Directive minds: How dynamics shapes cognition,” in J. Stewart, O. Gapenne and E. A. Di Paolo (Eds.), Enaction: Toward a New Paradigm for Cognitive Science (Cambridge, MA: MIT Press) 219–243. 10.7551/mitpress/9780262014601.003.0009

[B20] Feinberg T. E. (2012). Neuroontology, neurobiological naturalism, and consciousness: a challenge to scientific reduction and a solution. Phys. Life Rev. 9, 13–34. 10.1016/j.plrev.2011.10.019 22056393

[B21] Feinberg T. E. Mallatt J. M. (2016). The Ancient Origins of Consciousness: How the Brain Created Experience. Cambridge, MA: MIT Press. 10.7551/mitpress/10714.001.0001

[B22] Feinberg T. E. Mallatt J. M. (2018). Consciousness Demystified. Cambridge, MA: MIT Press. 10.7551/mitpress/11793.001.0001

[B23] Gallagher S. Zahavi D. (2008). The Phenomenological Mind: An Introduction to Philosophy of Mind and Cognitive Science (1st ed.). New York: Routledge. 10.4324/9780429319792-1

[B24] Ginsburg S. Jablonka E. (2019). The Evolution of the Sensitive Soul: Learning and the Origins of Consciousness Cambridge, MA: MIT Press. 10.7551/mitpress/11006.001.0001

[B25] Godfrey-Smith P. (2020). Metazoa: Animal Minds and the Birth of Consciousness. London: William Collins.

[B26] Grasso F. W. Basil J. A. (2009). The evolution of flexible behavioral repertoires in cephalopod molluscs. Brain Behav. Evol. 74, 231–245. 10.1159/000258669 20029186

[B27] Humphrey N. (1992). A History of the Mind: Evolution and the Birth of Consciousness. New York: Copernicus - Springer-Verlag. 10.1007/978-1-4419-8544-6

[B28] Irwin L. N. (2020). Renewed perspectives on the deep roots and broad distribution of animal consciousness. Front. Syst. Neurosci. 14, 57. 10.3389/fnsys.2020.00057 32903840 PMC7438986

[B29] Irwin L. N. Irwin B. A. (2020). Place and environment in the ongoing evolution of cognitive neuroscience. J. Cogn. Neurosci. 32, 1837–1850. 10.1162/jocn_a_01607 32662725

[B30] Jackson J. H. (1887). Remarks on evolution and dissolution of the nervous system. J. Mental Sci. 23, 25–48. 10.1192/bjp.33.141.25

[B31] Koch C. (2012). Consciousness: Confessions of a Romantic Reductionist. Cambridge, MA: MIT Press. 10.7551/mitpress/9367.001.0001

[B32] LeDoux J. (2019). The Deep History of Ourselves: The Four-Billion-Year Story of How We Got Conscious Brains. Viking, New York.

[B33] Mallatt J. Blatt M. R. Draguhn A. Robinson D. G. Taiz L. (2021). Debunking a myth: plant consciousness. Protoplasma. 258, 459–76. 10.1007/s00709-020-01579-w 33196907 PMC8052213

[B34] Mather J. A. (2008). Cephalopod consciousness: behavioural evidence. Conscious Cogn. 17, 37–48. 10.1016/j.concog.2006.11.006 17240163

[B35] Merker B. (2005). The liabilities of mobility: a selection pressure for the transition to consciousness in animal evolution. Conscious Cogn. 14, 89–114. 10.1016/S1053-8100(03)00002-3 15766892

[B36] Montemayor C. (2021). Types of consciousness: The diversity problem. Front. Syst. Neurosci. 15, 747797. 10.3389/fnsys.2021.747797 34880733 PMC8647661

[B37] Nieder A. Wagener L. Rinnert P. (2020). A neural correlate of sensory consciousness in a corvid bird. Science 369, 1626–1629. 10.1126/science.abb1447 32973028

[B38] Paul E. S. Sher S. Tamietto M. Winkielman P. Mendl M. T. (2020). Towards a comparative science of emotion: Affect and consciousness in humans and animals. Neurosci. Biobehav. Rev. 108, 749–770. 10.1016/j.neubiorev.2019.11.014 31778680 PMC6966324

[B39] Pennartz C. M. A. Farisco M. Evers K. (2019). Indicators and criteria of consciousness in animals and intelligent machines: An inside-out approach. Front. Syst. Neurosci. 13, 25. 10.3389/fnsys.2019.00025 31379521 PMC6660257

[B40] Putnam H. (1967). Psychological predicates. Art Mind Religion 1, 37–48.

[B41] Schnell A. K. Clayton N. S. (2021). Cephalopods: Ambassadors for rethinking cognition. Biochem. Biophys. Res. Commun. 564, 27–36. 10.1016/j.bbrc.2020.12.062 33390247

[B42] Seth A. K. (2021). Being You: A New Science of Consciousness. New York: Penguin Random House.

[B43] Sheets-Johnstone M. (1999). The Primacy of Movement. Amsterdam: John Benjamins Publishing. Vol. 14. 10.1075/aicr.14

[B44] Tononi G. Edelman G. M. (1998). Consciousness and complexity. Science 282, 1846–1851. 10.1126/science.282.5395.1846 9836628

[B45] Tononi G. Koch C. (2015). Consciousness: here, there and everywhere? Philos. Trans. R Soc. Lond B Biol. Sci. 370, 20140167. 10.1098/rstb.2014.0167 25823865 PMC4387509

[B46] Vallortigara G. (2020). The rose and the fly. A conjecture on the origin of consciousness. Biochem. Biophys. Res. Commun. 564, 170–174. 10.1016/j.bbrc.2020.11.005 33213842

[B47] Varela F. (1996). Neurophenomenology: A methodological remedy for the hard problem. J. Consciousness Stud. 3, 330–349.

[B48] Young J. (1964). A Model of the Brain. London: Oxford Univ Press.

